# Mechanical Properties of Fly Ash-Based Geopolymer Concrete Incorporation Nylon66 Fiber

**DOI:** 10.3390/ma15249050

**Published:** 2022-12-18

**Authors:** Muhd Hafizuddin Yazid, Meor Ahmad Faris, Mohd Mustafa Al Bakri Abdullah, Muhammad Shazril I. Ibrahim, Rafiza Abdul Razak, Dumitru Doru Burduhos Nergis, Diana Petronela Burduhos Nergis, Omrane Benjeddou, Khanh-Son Nguyen

**Affiliations:** 1 Center of Excellence Geopolymer & Green Technology (CEGeoGTech), Universiti Malaysia Perlis (UniMAP), Kangar 01000, Malaysia; 2 Faculty of Chemical Engineering & Technology, Universiti Malaysia Perlis (UniMAP), Kangar 01000, Malaysia; 3 Department of Civil Engineering, Faculty of Engineering, Universiti Malaya, Kuala Lumpur 50603, Malaysia; 4 Faculty of Materials Science and Engineering, Gheorghe Asachi Technical University of Iasi, 700050 Iasi, Romania; 5 Department of Civil Engineering, College of Engineering, Prince Sattam Bin Abdulaziz University, Alkharj 16273, Saudi Arabia; 6 Faculty of Materials Technology, Ho Chi Minh City University of Technology—HCMUT, Ho Chi Minh City 70000, Vietnam

**Keywords:** geopolymers, geopolymer concrete, polymer fiber reinforced geopolymers, interfacial bonding

## Abstract

This study was carried out to investigate the effect of the diamond-shaped Interlocking Chain Plastic Bead (ICPB) on fiber-reinforced fly ash-based geopolymer concrete. In this study, geopolymer concrete was produced using fly ash, NaOH, silicate, aggregate, and nylon66 fibers. Characterization of fly ash-based geopolymers (FGP) and fly ash-based geopolymer concrete (FRGPC) included chemical composition via XRF, functional group analysis via FTIR, compressive strength determination, flexural strength, density, slump test, and water absorption. The percentage of fiber volume added to FRGPC and FGP varied from 0% to 0.5%, and 1.5% to 2.0%. From the results obtained, it was found that ICBP fiber led to a negative result for FGP at 28 days but showed a better performance in FRGPC reinforced fiber at 28 and 90 days compared to plain geopolymer concrete. Meanwhile, NFRPGC showed that the optimum result was obtained with 0.5% of fiber addition due to the compressive strength performance at 28 days and 90 days, which were 67.7 MPa and 970.13 MPa, respectively. Similar results were observed for flexural strength, where 0.5% fiber addition resulted in the highest strength at 28 and 90 days (4.43 MPa and 4.99 MPa, respectively), and the strength performance began to decline after 0.5% fiber addition. According to the results of the slump test, an increase in fiber addition decreases the workability of geopolymer concrete. Density and water absorption, however, increase proportionally with the amount of fiber added. Therefore, diamond-shaped ICPB fiber in geopolymer concrete exhibits superior compressive and flexural strength.

## 1. Introduction

Traditional Portland cement (OPC) is regarded as the most widely used construction material in the world for the production of mortars. Large amounts of energy derived from the combustion of fossil fuels are used in the production of OPC, resulting in the emission of greenhouse gases, such as carbon dioxide (CO_2_). According to earlier research, 1.5 metric tons of raw materials are required to produce one metric ton of cement, resulting in the emission of 0.8 metric tons of CO_2_ into the environment [1].

Many studies have been conducted in an effort to reduce the OPC contents in concrete mixtures by partially or entirely substituting the OPC with a mineral addition or industrial by-product such as fly ash, slag, or silica fume in order to reduce CO_2_ emissions [2]. Due to the substitution of aluminosilicate materials, geopolymers have been introduced as alternatives to OPC in the construction field. Geopolymers can be made from any raw materials that have a high silica (SiO_2_) and alumina (Al_2_O_3_) composition as their main constituents, can react with a concentrated alkaline solution, and have thermal energy for curing to speed up the reactions [3].

One of the commonly used aluminosilicate materials is fly ash. Fly ash is a by-product that is produced from burning anthracite or bituminous coal. Fly ash is widely available over the world, possesses pozzolanic properties, and is high in alumina and silicate, but its application has been limited so far. Despite the fact that coal-burning power plants are environmentally harmful, the amount of energy produced by them is increasing due to the huge global supply of high-quality coal and the low cost of energy produced from these sources [4,5,6].

Due to the material’s high compressive strength and low tensile strength, geopolymer has been shown to possess mechanical properties similar to those of hardened cement (brittle). Fiber reinforced concrete (FRC), also known as conventional concrete, is made by randomly adding tiny fibers to the concrete mixture to increase its brittleness. If a crack develops in plain concrete while it is being loaded, it spreads quickly and results in a loss of load carrying capacity. In contrast, with FRC, the break is intercepted by the fibers scattered throughout the matrix, causing it to slow down and even come to a stop. This process, known as the “crack bridging effect”, increases the toughness of the concrete and maintains its capability of supporting a load even after the first crack appears [2].

The type of fiber, fiber content, bonding strength between the fiber and matrix, and mechanical properties of the fiber are significant in improving the mechanical qualities of geopolymer concrete (GPC). Steel fiber (SF) and polypropylene fiber (PF) are the two most common forms of fiber [7]. Metallic fibers frequently enhance flexural strength due to their higher stiffness, whereas non-metallic fibers regulate plastic matrix shrinkage due to their larger aspect ratio and surface contact area [8]. PF is believed to improve the performance of concrete owing to its high impact resistance, greater strain to failure, fine crack-free finish, increased water permeability resistance, and subsequently improved durability.

On the other hand, fibers from a variety of materials, including metal-based fibers such as steel and stainless-steel alloys; carbon-based fibers such as PAN rayon and mesophase pitch; synthetic fibers such as polyvinyl alcohol, polypropylene, and polyethylene; natural fibers such as jute, sisal, bamboo, and coconut; and inorganic fibers such as silica and basalt, are frequently used in composite materials [9].

The high strength, high modulus fibers, such as steel, glass, asbestos, carbon, and etc., are primarily used to acquire superior strain hardening after peak load, fracture toughness, and resistance to fatigue/thermal shocks, whereas the low modulus, high elongation fibers, such as nylon, polypropylene, PET (Polyethylene terephthalate), polyester, and shredded tire wastes, are potentially used in, but not limited to, enhancement of energy absorbed [10].

The most common steel and polypropylene fibers employed in nylon fiber research were quite few. A synthetic substance is nylon. Nylon is a smooth, thermoplastic substance that may be melted and processed into a variety of “films, fibers, or forms”. Nylon fiber was chosen because it has excellent hardness, resilience, and durability qualities. It is also easily available in a wide range of colors, can be dyed, is resistant to soil and filth, has high abrasion capabilities, and can be cut into various cross sections. Nylon is resistant to a range of materials, hydrophilic, heat stable, and generally inactive. After the first fracture, nylon is most effective in increasing concrete’s load-bearing capacity, flexural toughness, and impact resistance [11].

The prospect of adding fibers as reinforcement to a geopolymer matrix is therefore the subject of investigation. These materials’ flexural strength and fracture toughness should both be strengthened by the addition of fibers, as well as the energy that the geopolymer can take before suffering damage. Strengthening geopolymers with short fibers is particularly effective because of how easily they can be dispersed. A fracture becomes more ductile and less brittle as fibers are added. The material’s cracks are less numerous, and they are smaller in size, with a maximum crack width. This is especially true of microcracks, which are less likely to spread [12].

The main objective of this study is to investigate the strength of the fly ash geopolymer concrete reinforced fibers at two different curing times, which are 28 days and 90 days. The nylon66 fiber has been introduced in this study due to its good properties. This study also focuses on the effect of interlocking plastic bead (diamond end shape) toward the geopolymer concrete strength properties.

## 2. Materials and Methods

### Preparation of NFRGC

In this study, Nylon66 Fiber Reinforcement Geopolymer Concrete (NFRGC) was used in the formation of geopolymers alongside other materials, including fly ash, alkali activator, and aggregates. Sodium silicate (Na_2_SiO_3_) and sodium hydroxide (NaOH) were combined to create an alkali activator with a ratio of 2.5. 12M of NaOH concentration was used in this research, which was achieved by diluting the NaOH pellet in distilled water at the desired concentration. Meanwhile, the ratio of fly ash to an alkali activator was fixed at 2.0. All of the selected ratios for the formation of fly ash geopolymers in Table 1 and Table 2 were based on the previous findings [13].

The fly ash class C used in this study was taken from the plant Manjung, Perak, Malaysia. There are two types of aggregates used in this study: fine and coarse. River sand was used as fine aggregate, and granite was used as coarse aggregate, with sizes of 4.7 mm and 20 mm, respectively. The combination ratio for both aggregates is 60% coarse and 40% fine by weight. Meanwhile, the ratio between geopolymers and aggregate is 40% geopolymers and 60% aggregate.

**Figure 1:**
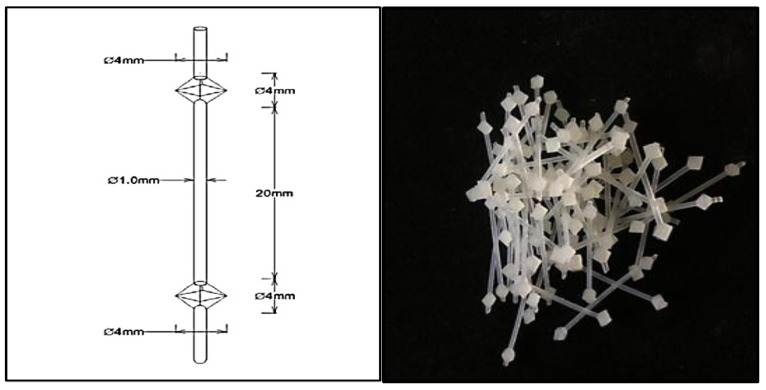
The plastic fiber and specification.

Fly ash and alkali activators are mixed at a ratio of 2.0 to create the geopolymer paste. Nylon66 fibers of diamond form were used in this experiment, which involved Interlocking Chain Plastic Beads (ICPB). The volume fraction of samples with compressive and flexural strengths of 0%, 0.5%, 1.0%, 1.5%, and 2.0% was used to determine the amount of nylon66 fibers to add to the geopolymer concrete mixture. Addition of 0%, 0.5%, 1.0%, 1.5%, and 2.0% by volume were tested for compressive strength testing. Additional information on Nylon fiber specification used in the production of NFRGC is summarized in Figure 1.

To create the required and precise shape and dimensions of the plastic bead, a unique mold was created. The beads’ shape was created using the plastic injection molding process. With controlled speed and pressure, a molten nylon66 resin mixture colored with white was injected into the mold. The substance was then freed from the mold after cooling and taking on the appropriate form. The procedure was repeated in order to obtain more units. Six (6) different beads per set make up the linked plastic beads. They had two and three-bead systems cut off. This study does not include a fiber-type processing step. The fiber type was applied in this study to investigate the effect of ICPB in geopolymer concrete, since studies on the diamond shape of ICPB are still limited. Nylon66 is noted as a polymer, and thus has poor bonding between matrix and fiber compared to metallic fibers, but excellent corrosion resistance. In this study, new virgin material was used instead of recycled material to reduce impurities.

There is no standard shape for aggregates because they all have unique forms; however, spherical and angular aggregates are the most popular and function well. Additionally, as aggregate shapes are inherently uneven after crushing and display similarities in terms of shape, size, and surface roughness, it is impossible to design or manufacture something that is precisely like an aggregate. Based on the situation, diamond-shaped beads were chosen as the form for the beads. The diamond shape is both round and slightly angular.

The NFRGC samples were cast in (100 mm × 100 mm × 100 mm) and (500 mm × 100 mm × 100 mm) molds for physical (workability, density, and water absorption) and mechanical (compressive and flexural) testing. Following a 24 h curing period, samples were removed from the mold and allowed to cure for 28 and 90 days at room temperature.

A slump test was used to evaluate the NFRGC’s workability. The ASTM C143 guidelines were followed for performing the slump test. After mixing, three layers of newly created geopolymer concrete were poured into a slump cone. Twenty-five tamping rod strokes were used to compress each layer. From the cone’s top, fresher NFRGC was scraped off. The freshly constructed NFRGC was then immediately raised vertically to eliminate the concrete cone’s workability. The slump was calculated by determining the separation between the top of the slump cone and the original center, which had been shifted, of the top surface of the new NFRGC.

A density test was conducted on the 28-day sample. A sample was submerged in water at room temperature for 24 h. In a water tank, the NFRGC sample was positioned apart from one another without touching. The top of the sample surface was no more than 150 mm relative to the still water line. To guarantee there was a 3 mm space between the sample and the bottom of the water container, the immersed sample was set on a wire mesh.

The 24 h immersed sample of NFRGC was weighed and recorded (Wi). The sample was then taken out of the water tank and left to dry for one minute. A moist towel was used to remove any apparent water from the sample’s surface. Afterwards, the sample was weighed and recorded as being saturated (Ws). After that, the sample was dried for 24 h at 110 °C in an oven. Following that, the dried sample was weighed and given a dried weight label (Wd).

A Universal Testing Machine (UTM) Automatic Max was used to determine the compressive strength of sample NFRGC in accordance with standard BS 1881-116. (Instron, 5569, Norwood, MA, USA). This testing was done on samples that were cured for 28 and 90 days at room temperature. A load speed adjustment of 0.1 kN/s was made.

The flexural test was carried out to gauge the sample’s flexural strength. Using the UTM model Automatic Max, the sample was put through a 4-point bending test (Instron, 5569, Norwood, MA, USA). The testing was carried out according to ASTM C1018. This study used a constant deflection rate that ranged from 0.05 to 0.10 mm/min. The lower and top supports were 300 mm and 100 mm in height, respectively. After being cured at room temperature for 28 and 90 days, the sample was examined.

## 3. Results and Discussion

### 3.1. Chemical Composition

Table 3 provides a summary of the chemical composition of the fly ash used to make geopolymer concrete with both types of fibers. There are five major elements that contribute to the properties of geopolymers, comprising ${\mathrm{SiO}}_{2}$, CaO, ${\mathrm{Fe}}_{2}O_{3}$,${\;\mathrm{Al}}_{2}O_{3}$ and MgO. It is worth noting that fly ash is composed of silicon oxide, aluminum oxide, iron oxides, and other minor oxides. Major components including SiO_2_ and Al_2_O_3_ contribute almost 90% of the total weight of fly ash. Meanwhile, Fe_2_O_3_ content is less than 5% of the total weight of fly ash. From Table 3, the total composition of SiO_2_ and Al_2_O_3_ are 43.9%, followed by CaO with 22.30%, Fe_2_O_3_ with 22.99%, and MgO with less than 1%. According to the chemical composition obtained, the fly ash used in this study was classified as Class C fly ash [14]. In addition, the fly ash used meets the basic requirements for a source of material to be used as a precursor due to its high Si and Al content, which is significant for creating geopolymer bonds.

Si-O-Al appeared as one of the most significant linkages that affected the strength of the geopolymer, and the combination of Si and Al maps demonstrated how it formed. The geopolymer typically has a favorable setting time due to being high in calcium (Ca) content. Although there was a significant difference in the amount of Ca in the two geopolymers, it was discovered that the strength growth was gradual. Meanwhile, increasing curing temperature and time resulted in increased strength. The presence of Si and Al components in geopolymer composites influences strength development because more geopolymer chains are formed, which strengthens the geopolymer composite materials. The majority of the geopolymer’s basic structure is made up of Si-O-Al, demonstrating the importance of Si and Al components in producing strong development. The presence of Mg, however, slowed the geopolymer’s strength growth. This has disrupted the Ca-Si-O-backbone Al structure, reducing the geopolymer’s ability to produce strength. In addition, due to the development of hydrotalcite group phases and a decrease in the amount of readily available Al element, appropriate Ca enables the formation of low Al C-(A)-S-H.

### 3.2. FTIR Spectera

**Figure 2:**
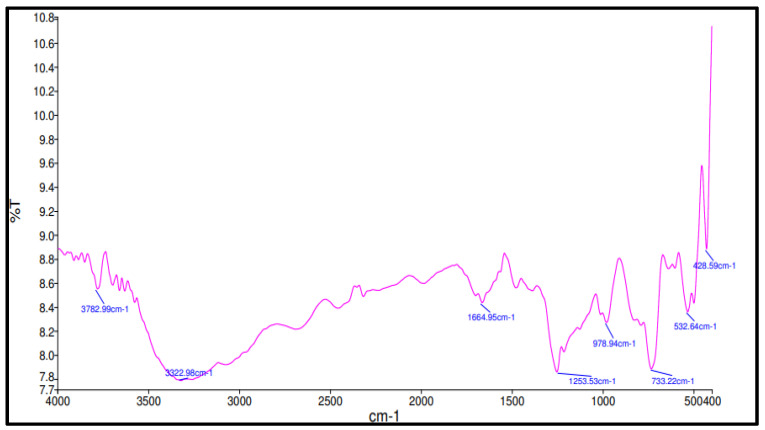
FTIR spectrum of fly ash.

The infrared analysis spectra of the applied fly ash are shown in Figure 2. The figure shows several peaks at 428 ${\mathrm{cm}}^{-1}$, 532.64 ${\mathrm{cm}}^{-1}$, 733.22 ${\mathrm{cm}}^{-1}$, 1253.53 ${\mathrm{cm}}^{-1}$, 1663.95${\;\mathrm{cm}}^{-1}$, 3222.98${\;\mathrm{cm}}^{-1}$, and 3782 ${\mathrm{cm}}^{-1}$. Absorption bands at 733.22${\;\mathrm{cm}}^{-1}$, 532.64 ${\mathrm{cm}}^{-1}$, and 428.59${\;\mathrm{cm}}^{-1}$ were labelled as O Si O links in quartz, and Si O and Al O bonds in zeolite frameworks, and the band surrounding 1000−960 cm−1 represents bonds of Si–O–T (T is tetrahedral Si or Al) of the geopolymer gel. Absorption bands at regions at 450 ${\mathrm{cm}}^{-1}$ can represent Si–O–Al linkage; Si O bond characterizes to bending vibration at 400−500 ${\mathrm{cm}}^{-1}$, and the stretching vibration at 800−1000 ${\mathrm{cm}}^{-1}$. Although, absorption bands in regions at 980 ${\mathrm{cm}}^{-1}$ can be related to O–Si–O bond bending vibration, or symmetric stretching vibrations of the Si–O–Si (Al) bridge [15,16].

The asymmetric stretching vibrations of the silicon tetrahedral (SiO4-4) found in the chain structure of the Si-O terminal bonds can also be attributed to several additional bands found in regions around 1253.53 ${\mathrm{cm}}^{-1}$ [17]. Meanwhile, the stretching vibration of O-H and H-O-H due to water and silanol group occurs within a range of 3222.98${\;\mathrm{cm}}^{-1}$ to 3782 ${\mathrm{cm}}^{-1}$. This indicates a stretching vibration of O-H and H-O-H from 82 water molecules which are weakly bonded that appear at the surface, or are trapped in a large cavity inside the geopolymer sample. In addition, a wavenumber of 1664.95${\;\mathrm{cm}}^{-1}$ represents bending vibration of H-O-H. 

**Figure 3:**
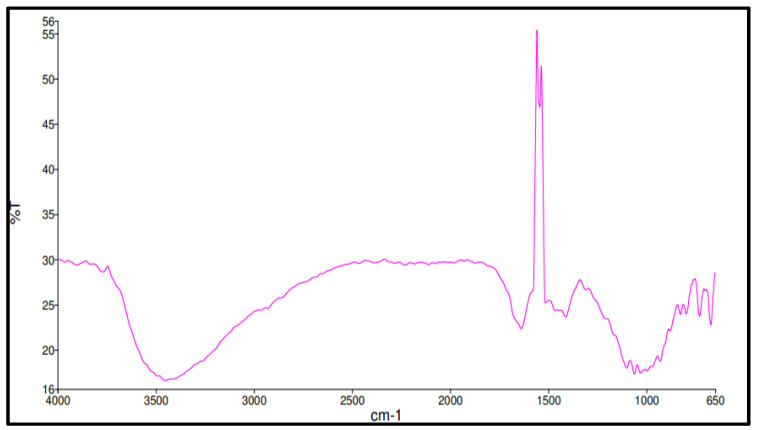
FTIR spectrum of geopolymers.

Meanwhile, infrared analysis spectra for the geopolymer concrete are illustrated in Figure 3. The result shows observation at peak 3775.37${\;\mathrm{cm}}^{-1}$, 3454.07${\;\mathrm{cm}}^{-1}$, 1638.68${\;\mathrm{cm}}^{-1}$, 1544.29${\;\mathrm{cm}}^{-1}$, 1411.56${\;\mathrm{cm}}^{-1}$, 1062.90${\;\mathrm{cm}}^{-1}$, and 671.18${\;\mathrm{cm}}^{-1}.$

The intensity of absorption bands at 671.18$\;{\mathrm{cm}}^{-1}$ is connected to the stretching vibration of the Si-O-Si symmetry and the bending vibration of the O-Si-O bonds (Al) [17]. The size of these bands is due to the material being amorphous. There is also a vibration band for the stretching of Si-O-A at 1062.90 ${\mathrm{cm}}^{-1}$. The Si-O-Al was determined by the peaks found between 700 and 1100 ${\mathrm{cm}}^{-1}$ [18]. In the peak from 1400 to 1450 ${\;\mathrm{cm}}^{-1}$ it was noticed the temperature rose to 1000 °C. As the peak shifted to 1411.56$\;{\mathrm{cm}}^{-1}$, the strength of the composite decreased. The band at 1411.56 ${\mathrm{cm}}^{-1}$ displays the feature of the asymmetric O-C-O stretching mode, which shows the existence of sodium carbonate due to the interaction between too much sodium and ambient carbon dioxide [19].

Three bands located at 1638.68${\;\mathrm{cm}}^{-1}$, 3375.37${\;\mathrm{cm}}^{-1}$, and 3454.07${\;\mathrm{cm}}^{-1}$ were associated with the water molecules. As a result of the inclusion of nanoparticles, the overall spectra also demonstrated an increase in the intensity of the Si-O-Al band, suggesting a rise in the quantity of N-A-S-H gel [20]. Simultaneously, the frequency moved to a higher wavenumber at 1544.29$\;{\mathrm{cm}}^{-1}$ as rising solid/liquid ratios, which suggested calcite vibration. Calcite and amorphous silica were produced when tobermorite decalcified, which caused the wavenumber to change [21]. Peak calcium-based component intensity demonstrated the dominance of high strength geopolymer structure.

### 3.3. Compressive Strength

**Figure 4:**
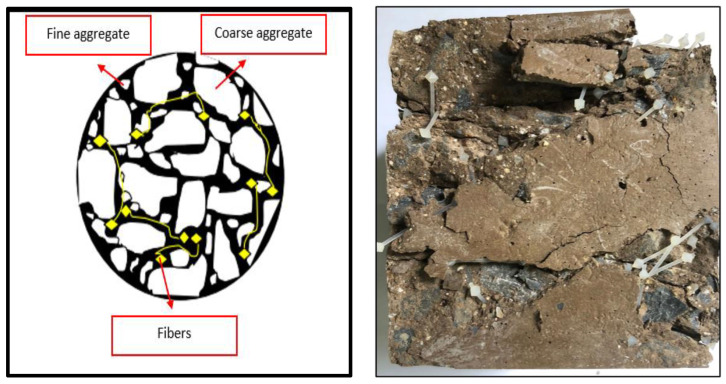
The chained interlocking plastic beads schematic diagram illustrated.

Fiber reinforced geopolymers at 28 days, as well as Geopolymer concrete with nylon66 fiber (NF) reinforcement’s compressive strength for both samples at 28 and 90 days. The compressive strength of geopolymer concrete appears to increase with plastic fiber addition, up to a maximum value at 0.50% of fiber addition. This is because nylon66 fibers, which restrict cracks from spreading during compression loads, and linked interlocking plastic beads act as reinforcing agents by interlocking with each other in the aggregate skeleton. The main strategy used in this study is to fill the spaces between the fine and coarse aggregate with beads to give them an interlocking strength using a linked plastic system, as illustrated in the schematic picture in Figure 4. The weak interfacial connection between the matrix and the fiber caused by hydrophobic surface characteristics was significantly improved by the linked interlocking plastic beads. As a result of the nylon66 fibers’ contribution, the geopolymer binder slid out of the nylon66 fibers’ diamond-shaped ends with greater resistance than the straight fiber without anchorage.

**Figure 5:**
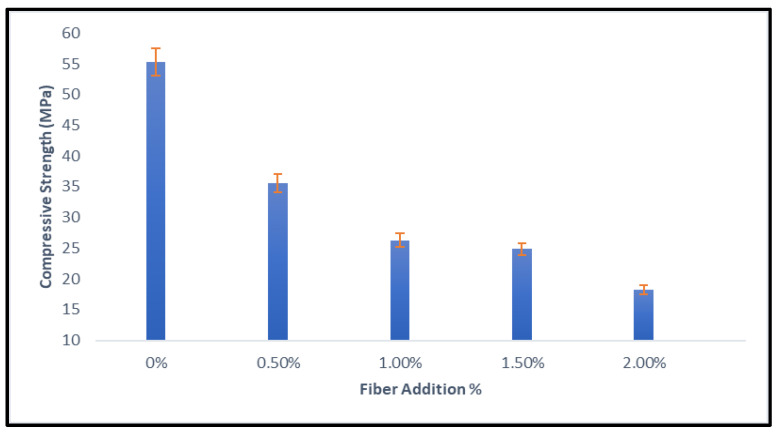
Compressive strength of geopolymers.

The compressive strength of geopolymers with fiber addition is depicted in Figure 5. The results demonstrate that fly ash geopolymers without nylon66 fiber addition have higher strengths, and the strength starts to decrease with the inclusion of nylon66 fiber. Even though the reduction in strength is about 35% at 0.50v% fiber addition, the strength obtained is still notably higher (35.59 MPa). According to the results, adding nylon66 fiber or linked interlocking plastic beads did not improve the compressive strength properties of geopolymers. The interfacial connection between the matrix and fiber is believed to be weak due to the smooth or hydrophobic surfaces of the polymers, and the fiber cannot inhibit the spread of cracks in geopolymers. However, some regions in the geopolymer matrix that include nylon66 fibers are believed to fill the voids between fly ash particles with beads to give them an interlocking strength and contribute to good strength. Insertion of fiber greater than 0.50% disturbs the CASH bonding in the geopolymer matrix and diminishes its compressive strength. According Patrycya et al. [22], the optimum result obtained on geopolymers reinforced with hooked-end steel fiber and melamine fiber was circa 0.5% amount of fiber by weight. The result shows that plain GPC is 40 MPa; steel fiber 0.5% is 40 MPa, and 1.0% is 39 MPa; and melamine fiber 0.5% is 50 MPa, and 1.0% is 45 MPa. Melamine fiber has better resistance to force. Based on the research, fiber shape gave an effect to the compressive strength on geopolymers; hooked-end type steel fiber held the matrix with greater force during crack propagation [23]. The schematic function of fiber that was used in this study for the geopolymer concrete was illustrated previously in Figure 4. The addition of nylon66 fibers’ (ICPB) diamond shape on reinforced geopolymers was intended to investigate the effect on the compressive strength between GP and GPC. 

**Figure 6:**
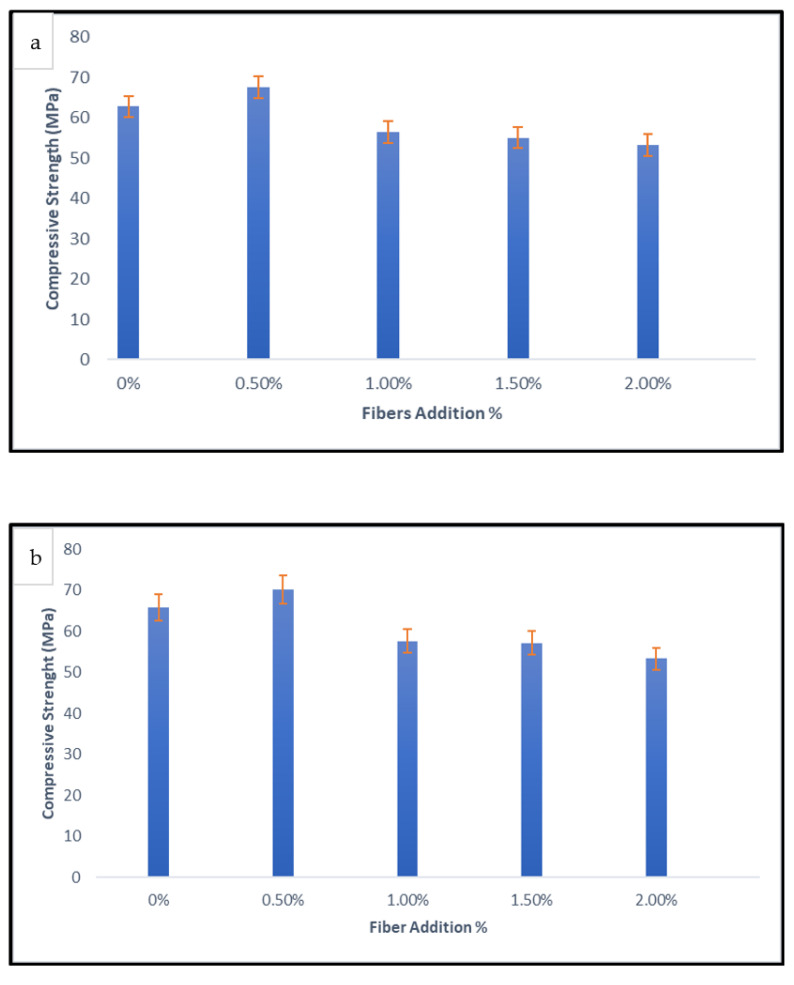
(**a**) Compressive strength of NFRGC for 30 days and (**b**) Compressive strength of NFRGC for 90 days.

Figure 6a depicts the compressive strength of Nylon66 Fiber Reinforced Geopolymer Concrete (NFRGC) after 28 days of room temperature curing. It was discovered that adding 0.5% of fiber resulted in a higher compressive strength with a value of 67.6 MPa, which then decreased to 53.3 MPa with the addition of fiber at 2.0%. Geopolymer concrete has a high compressive strength, and suitable fiber addition as well as fiber type were discovered to increase properties depending on the application. Chained interlocking plastic-bead fibers increase the strength of NFRGC as compared to geopolymers. This is due to the capacity of Nylon66 fibers to delay the spread of cracks during compression loads. This can be attributed to RTS fiber’s high stiffness and hydrophilicity, which allow it to absorb more energy and form a strong fiber-matrix interaction [23,24].

Since Nylon66 fiber has a lower young modulus than steel fiber, increasing fiber addition in geopolymers results in a negative trend in concrete. As additional fiber is added, the compressive strength decreases while the toughness increases due to the higher elasticity of nylon66 fiber over geopolymer concrete. Fiber length influences compressive strength or toughness strength, and research has shown that short fiber is ideal for these qualities as well as to avoid microcrack propagation. Composites with an irregular internal structure resulted in reduced compressive strengths and variable compressive behaviour. As a result, the incorporation of metallic fibers could improve the mechanical properties of GPC, whilst the high fraction of nylon66 fibers in GPC could lower its mechanical performance. The substantial standard deviations of the findings of the hybrid replacement series with more fiber made this apparent. Meanwhile, Figure 6b illustrates the NFRGC’s compressive strength after 90 days.

The compressive strength of NFRGC is affected by the curing period. After 90 days, the compressive strength of the NFRGC has increased in comparison to 28 days. After 90 days, the compressive strength increased to 70.13 MPa, from 67.6 MPa at 28 days, with the addition of 0.5% fiber. However, once the fiber inclusion exceeds 0.5%, the compressive strength of geopolymers decreases. When the NF volume exceeds 0.5%, the decrease in compressive strength is primarily due to the difficulty of fiber distribution, especially in large volume fractions, which is caused by poor workability and inadequate compaction. In contrast, NFRGC with the lowest nylon66 fiber concentration achieved the highest compressive strength, owing to the mechanical properties of nylon66 fiber. The fibers in concrete contributed to energy dissipation via the bridging effect of their shape and mechanical properties. The frictional bonding that develops as a result of the resistance to pulling out the nylon66 fibers, caused by friction between the fibers and the geopolymer matrix, contribute to the NFRGC’s high strength [25].

In addition, as the fiber content increased to 1%, compressive strength decreased substantially from 70.13 to 57.5 MPa. This is believed to be attributed to the material’s poor compaction and significant voids. Due to the material’s high degree of flexibility, high volume fractions of Nylon66 fiber make compaction difficult, resulting in a loose and porous geopolymer matrix. The relative density of fiber-reinforced geopolymer composites, on the other hand, was decreased by adding more fibers. This is due to the fact that the air bubbles caused by imperfect vibration in the composite products caused the relative density to increase. This condition hinders the consolidation of the fresh mixture, and even the long exterior vibrations are ineffective at compacting the concrete. As observed, an increase in fiber content above 2% has a negative impact on composite density.

The compressive strength of fiber reinforced concrete increased initially and subsequently declined as the nylon66 fiber content grew from 0% to 2%, with a 0.5% optimal point where the internal structure of geopolymer concrete was considerably enhanced. The main factor causing the decline in compressive strength when the NF percentages are more than 0.5% is the difficulty in dispersing fiber, especially in large volume fractions, which contributes to poor workability and insufficient compaction [26].

Judging by previous work, there are no studies focusing on Nylon as fiber in concrete. However, other types of fiber such as PP and PF were the guidance in this research. According to Wang et al. [27] the compressive strength of polypropylene (PP) fiber reinforced geopolymer concrete with fiber length of 12 mm was observed to be slightly higher than that of 3 mm. The fiber type was straight fibers. Longer fibers performed better in terms of bridge effects because of the increased contact area between them and the geopolymer concrete, which led to a greater frictional force. In comparison to shorter strands, longer fiber could connect more air spaces. This research shows that effect of length contributes to the contact area and helps improve the bonding of polymer fibers and geopolymers. Compared to our study using long fiber, short fiber can also be improved by size and shape.

Piti et al.’s [2] study used the PF crimped type in fiber reinforced geopolymers, based on the compressive strength result that 0.5% fiber content is the optimum result. The plain geopolymer’s strength was 40.08 MPa, and the strength improved to 47.0 MPa after being reinforced by fibers at 0.5%. This illustrated that the trend of compressive strength was decreased to 34.49 MPa at 1.0% and so on.

According to Ranjbar et al.’s [15] investigation on the mechanisms of interfacial bond in steel and polypropylene reinforced geopolymer composite, after curing PF reinforced geopolymers for 56 days, the compressive strength of the plain geopolymers was the highest compared to others with fiber added. They illustrated that 0.5% content was the best, with 45 MPa compressive strength, compared with 1%, 2%, 3%, and 4% contents.

The compression results between 28 and 90 days followed the same pattern as the optimal result, indicating that a fiber content of 0.5% is the best result. The 90-day outcome was somewhat enhanced due to the geopolymers themselves. Curing time and temperature are significant factors in the hydration process of geopolymerization, with higher temperatures accelerating the hydration process and contributing to the geopolymer’s high strength. An extended curing period, however, influences the performance of geopolymers. In this instance, curing time enhances the compressive strength of geopolymers. The addition of NF reinforced in fly ash-based geopolymers progressively causes a geopolymer bonding reaction in the NFRGC, and the interlocking between the fiber, aggregate, and geopolymer matrix gets better with time.

**Figure 7:**
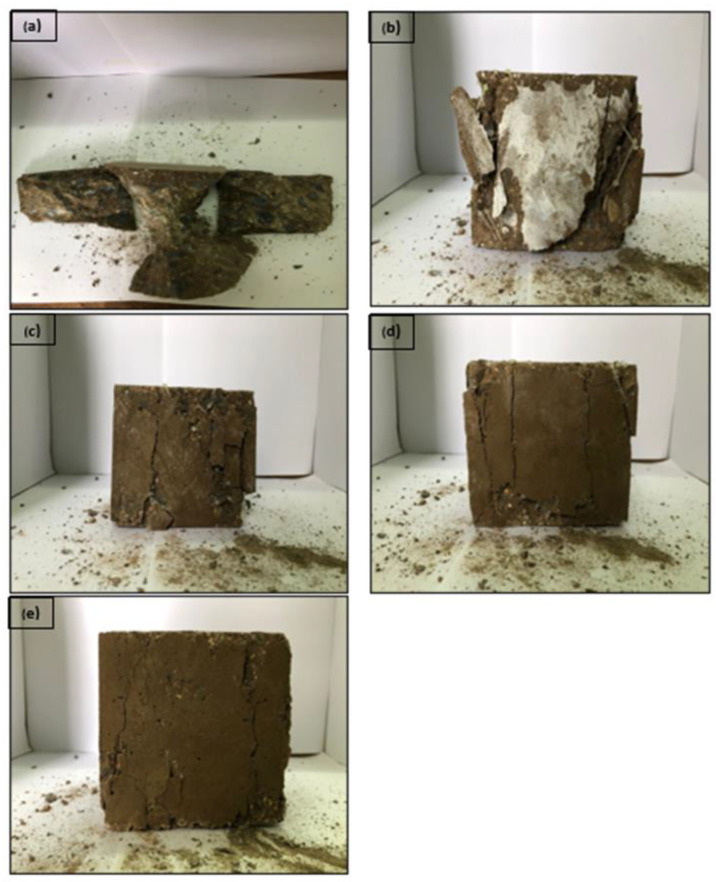
Crack pattern of geopolymer concrete (**a**) without fiber, (**b**) 0.5%, (**c**) 1.0%, (**d**) 1.5%, and 2.0% (**e**) with a schematic of failure mode.

The failure mode of the NFRGC cube when compressed is shown in Figure 7. All NFRGC specimens maintained their forms with little debris even after compression-induced failure, which is often characterized by evident large fissures.

Figure 7a shows the geopolymer concrete breaks into parts due to the brittle properties of geopolymer concrete. The fibers provided greater energy for resisting tensile tension in the cube, which prevented tensile fracture growth. Without fiber, geopolymer concrete can withstand the high load of energy. Addition of nylon66 fibers make the major crack propagation directly occur without displaying signs of crack growth prior to breakage.

In comparison to geopolymer concrete without fiber addition, 0.5% has the highest compressive strength of all results, despite having significant fracture propagation. The addition of fiber improves geopolymers’ ability to absorb energy, and the interlocking plastic beads aid in limiting crack propagation, resulting in the major crack spreading from the minor crack after 0.5% fiber was added. The inclusion of more fibers reduces compressive strength; however, crack propagation was decreased from major to minor due to the energy supplied into the fibers during the compression test to slow or stop the crack growth. The tensile strength was only slightly different from the value reported in the work of Arsalan et al. [25], which included NF as a fiber addition to the concrete mix. In addition to selecting the proper fiber fraction, geopolymer concrete must also have equally distributed fibers in order to achieve the desired amount of strength.

During the compressive test, the greater fiber volume controlled the development of cracks. Geopolymer density decreases as fiber volume increases, whereas compressive strength and toughness increase. With the interlocking chain fiber, it is feasible to reduce energy transmission from the geopolymer concrete itself. It exchanges energy with the fiber to slow the spread of cracks. Nylon66 fiber can restrict the spread of cracks in geopolymer concrete, as shown in Figure 7e. It exhibits the symptoms of material breakdown as the crack spreads.

### 3.4. Flexural Strength

Figures below illustrate the flexural strength of geopolymer concrete and NFRGC with fiber addition after 28 and 90 days of curing, respectively. The flexural strength of GPC was observed to be enhanced with fiber added compared to plain GPC, and this improvement increased as the volume percent of fiber in the GPC increased.

**Figure 8:**
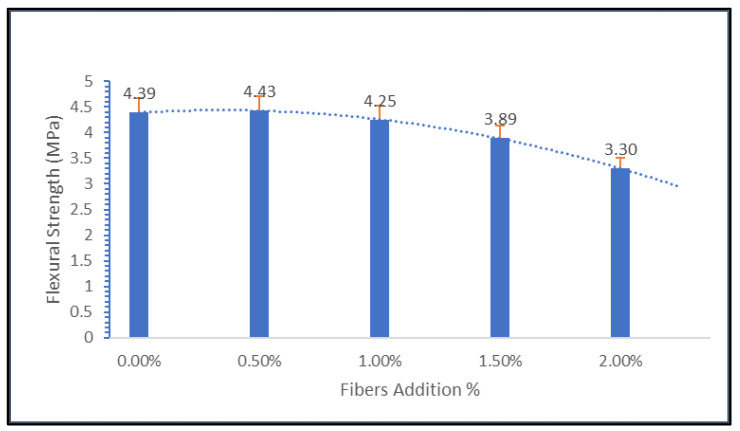
Flexural strength for 28 days.

From Figure 8, it was found that the inclusion of geopolymers led to an increment of the flexural strength of a concrete to a maximum of 0.5%, or 4.43 MPa. Meanwhile, normal geopolymer concrete without fiber addition exhibits a flexural strength of 4.39 MPa. Furthermore, when the nylon66 content exceeded 0.5%, the flexural strength began to decline. This was due to the samples’ poor workability when nylon66 fibers were added in large quantities. It is believed that this poor workability has an impact on the distribution of nylon66 fibers. As a result, when loading was applied, the absorption capacity inside the sample was unbalanced, thus causing crack formation.

Due to the limited availability of fiber, none of the three varieties have the same forms, surface smoothness, or aspect ratio, making direct comparisons using normalized or deleted measurements of the same level difficult. The tensile strength of the fiber has the greatest influence on post-crack behavior, and these factors only have an impact on the first fracture load. Furthermore, for each type of fiber used, various fiber volume fractions are generated, taking into account cost, density, and fiber dispersion in the concrete mix.

**Figure 9:**
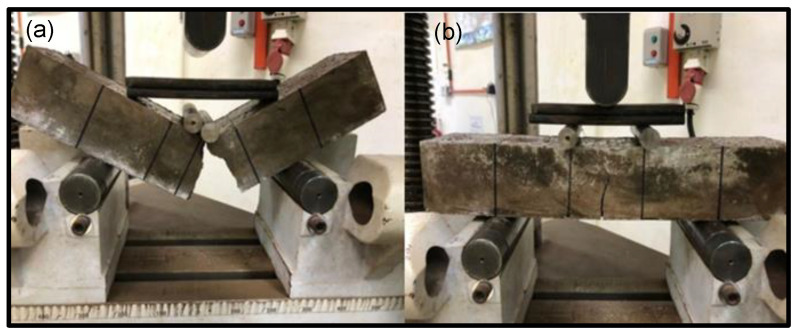
Shows the sample of bending test (**a**) geopolymer concrete without fiber addition and (**b**) with fiber addition.

As shown in Figure 9, the nylon66 fiber played a role in enhancing flexural strength by inhibiting crack propagation during flexural testing by bridging at the crack region. With the addition of Nylon66 fibers, the sample was able to sustain a larger flexural force prior to complete failure. Photographic observation of the crack and final fracture in NFRGC and geopolymer concrete with various fiber additions is shown in Figure 9.

The fiber failure mode demonstrates which type of feature dominates the flexural performance of geopolymer concrete. The majority of fibers do not draw out. All fibers are not extracted, particularly in the NFRGC. In this case, the binding property between the fiber and the concrete significantly influences how effectively the structure bends. The majority of the fibers rip apart at the fracture surface, indicating that fiber tensile behavior has a major influence on reinforced concrete flexural performance.

With an increase in the percentage of fiber volume, the number of fibers spread over the fracture surface increases, and the post-cracking performance is also enhanced. Fracture toughness, also known as post-crack performance, is expressed by the energy absorbed by a sample during deformation and failure.

**Figure 10:**
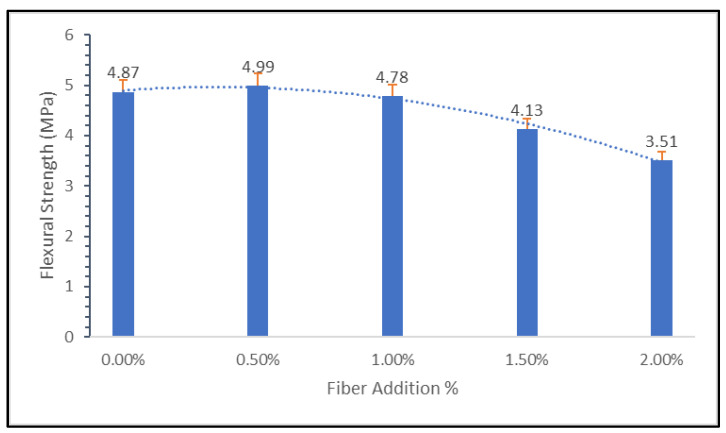
Flexural strength for 90 days.

Figure 10 shows that until the addition of 0.5%, the flexural strength of the geopolymer concrete increases to 4.99 MPa. Meanwhile, the plain geopolymer concrete without fiber addition has a flexural strength of 4.87 MPa. Flexural performance decreases when nylon66 fiber addition exceeds 0.5%. This is due to the samples’ poor workability when substantial amounts of Nylon66 fibers are introduced. It is believed that the low workability affects the distribution of nylon66 fibers. As a result, when loading is applied, the absorption capacity within the sample is unbalanced. The sample frequently cracks due to the fewer fibers available. The results of the comparison between 28 and 90 days show that the curing period is the most important factor in the development of the flexural strength.

Results for 90 days are better than those for 30 days since geopolymers are still hydrating slowly at that point. The pattern matches the compressive strength result exactly. In comparison to 30 days, the geopolymerization at 90 days enhanced the geopolymers’ characteristics, leading to better microstructure properties. According to Figure 10, it was found that Nylon66 fiber improved the flexural strength of geopolymer concrete. Regardless of the fiber type, the improvement in first-crack strength was expected due to the increase in fiber volume fraction.

Moreover, the nylon66 fiber reinforced geopolymer concrete was noted to be primarily responsible for the fiber bridging effect. Therefore, the characteristics of strain hardening and the flexural strength may be adversely affected by an increase in fiber content due to the uneven geometry of fiber from the recycling process.

When the specimen was subjected to the bending load, the area between the two loading pins, where the flexural stress was at its highest, began to deform and split. Once the matrix’s bending strength was exceeded, the first crack in composite materials began to form. After that, the crack continued to spread until it reached a nylon66 fiber with a low rigidity. The fracture attempted to penetrate through the fiber at this point due to the flexural tension being applied, which caused it to elongate, rupture, or pull out.

According to Wang et al. [27], the fiber addition was found to significantly improve the flexural strength of PPRGPC. The percentage of addition was varied at 0.1%, 0.15%, and 2.0%, respectively. This study used the PP fiber straight type. According to the result obtained, plain GPC obtained a flexural strength of 4 MPa and the strength was increased to 4.3 MPa with 0.1% fiber addition. Meanwhile, the flexural strength started to decrease for fiber addition at 0.15% (4.1 MPa) and 2.0% (4 MPa).

The geopolymerization product that had adhered to the nylon66 fibers’ surface suggested that the binding strength might be high enough to activate this mechanism. The interfacial bond strength, in contrast, is weaker than the applied stress. By redistributing the localized stress, fiber bridging caused the specimens to develop many microcracks. Increases in ductility and post-cracking toughness were brought on by the ongoing process of microcrack development.

Flexural strength at 28 and 90 days yielded results with several decimals since there was little significant variation in strength. The result is 5 MPa to 3 MPa between 28 and 90 days. Flexural strength displays the improvement and gap strength for each fiber added for 0.5%, 1%, 1.5%, and 2.0% with numerous decimals. The fiber distribution inside the geopolymers cannot be controlled, which is a problem. 

### 3.5. Water Absorption

**Figure 11:**
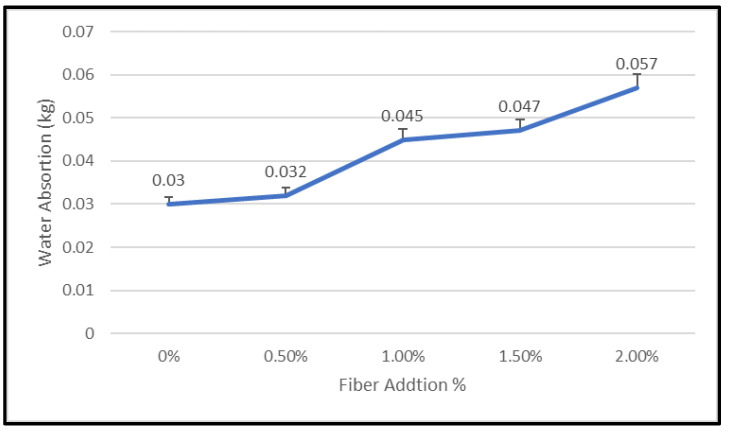
Water absorption of geopolymer concrete with addition of plastic fibers.

The results of the water absorption test are illustrated in Figure 11. With more fiber additions, the water absorption of geopolymer concrete increased. For geopolymer concrete, the nylon66 fibers with a 2.0% concentration have the maximum water absorption (0.057). This is because the workability reduced with the addition of nylon66 fibers as discovered in this study, which might cause an increase in the creation of pores. 

Permeability is a measure of how efficiently water, air, and other chemicals, such as chloride ions, can be absorbed by geopolymer concrete. Similar to OPC concrete, geopolymer concrete also contains pores that enable the absorption of particular compounds. Higher porosity leads to higher water absorption, which lowers the density of the concrete. Meanwhile, less porosity leads to a higher density of geopolymers, decreasing water absorption. Figure 11 shows the significant relation between water absorption and density, as well as how fiber addition appears to improve water absorption due to higher density. The weak interfacial interaction of Nylon66 fiber with the matrix could lead to the formation of a void, which would increase water absorption as fiber addition increased. Furthermore, as the amount of nylon66 fiber in geopolymer concrete increases, the degree of compaction in the mix decreases, encouraging the volume of air voids in the geopolymer concrete.

According to Jawad et al. [11], when using nylon fibers, water absorption is increased by 3–6%. As compared to samples of ordinary concrete, samples of concrete reinforced with nylon fibers absorb slightly more water. Improved connection between microchannels in the concrete’s outer surface and binder matrix may be to blame for this. Additionally, studies show that the addition of fibers improves concrete captivity and water absorption due to the lengthening of the microchannels in the microstructure.

This degradation would affect the performance of the geopolymer concrete’s fiber reinforcement, including its compressive strength, flexural properties, fiber matrix interfacial bonding, and durability against blasting. It is essential that geopolymer concrete has minimal water absorption for better performance. The geopolymer concrete samples obtained from this study have a high potential for corrosion resistance due to low water absorption and the use of fiber material. Water absorption was investigated by weighing the sample after it was removed from the water, since in NRGPC, it alters the qualities of fibers made with poor resistance to corrosion. Because nylon66 fiber has a low water absorption rate and is unaffected by corrosion, the amount of fiber used in this investigation was not measured. 

### 3.6. Slump Test

**Figure 12:**
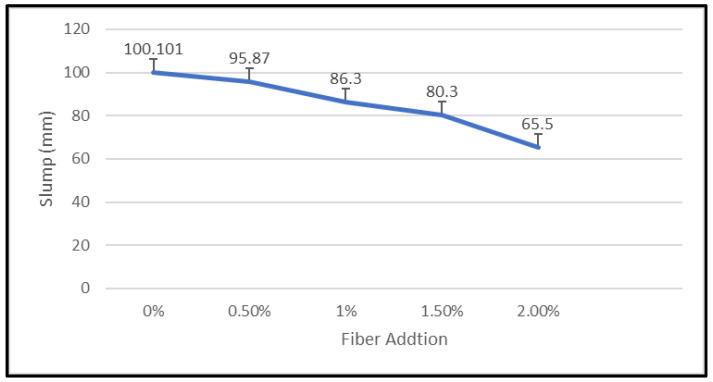
Slump of geopolymer concrete with addition of plastic fibers.

Using a standard slump cone, the slump was measured. In this study, the geopolymer concrete’s consistency and workability were assessed using the slump test. Figure 12 shows the decrease trend of workability for geopolymer concrete with addition of nylon66 fiber.

Figure 12 shows that the slump test result for geopolymer concrete without the inclusion of nylon66 fibers was 100.101 mm. The slump of geopolymer concrete with nylon66 fiber addition reduced with increasing additions of nylon66 fibers from 0% to 2%, which are 95.87 mm (0.5%), 86.3 mm (1%), 80.3 mm (1.5%), and 65.5 mm (2.0%). This has demonstrated that the presence of nylon66 fibers makes geopolymer concrete less workable. This finding suggests that the 65.5 to 100 mm range has low and medium workability.

This outcome also proved that the presence and addition of fibers significantly negatively impacted the workability of geopolymer concrete. This is due to increased friction between the geopolymer concrete matrix and fibers. The addition of more fibers causes the viscosity of new geopolymer concrete to increase because more binder is absorbed by the fibers’ higher surface area, resulting in low slump.

In addition, the fiber and coarse aggregate particles were noted to have compatible dimensions, which contribute to resisting the relative mobility of the latter. The flow of fresh geopolymer concrete was resisted in this condition, making it more difficult for coarse particles to move. This interlocking of fiber and aggregate is depicted in Figure 4. As a result, the difficulty of the relative movement between the coarse aggregates and the movement of the mixture increases with the number of fibers added. The mixture flows much more slowly and becomes less workable. The slump test was carried out using a slump cone and a mixture of fresh NFRGC, measuring the distance between the surface of the latter and the top of the slump to gauge the combination’s workability.

### 3.7. Density

**Figure 13:**
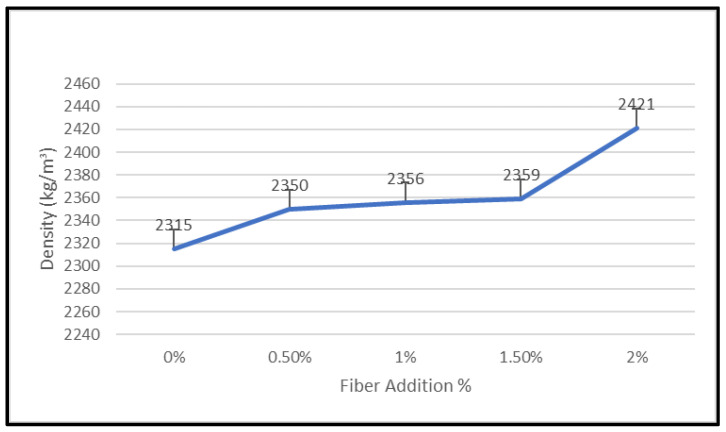
Density of plastic fibers against fiber addition.

Measured as mass per unit volume, density is the quantity of a substance. All samples were weighed after curing for 28 days at room temperature, and their masses were split by the mold’s 100 mm × 100 mm × 100 mm dimensions. The impact of adding fiber to geopolymer concrete’s density is seen in Figure 13.

According to the findings, 2421 $\mathrm{kg}/m^{3}$ was the density of the addition of 2% plastic fiber. In direct proportion to the addition of more nylon66 fibers, the geopolymer’s density rose. This result is illustrated by the range 2315–2421 $\mathrm{kg}/m^{3}$ after fiber was added. The results show that the density of GPC and NFRGC increase 4% at 2% fiber added. 

The change in density value with the addition of fiber in geopolymer concrete does not reveal any discernible trend in which the density rises and then somehow falls with the addition of a particular fiber. If the substitute fibers have nearly equal specific gravities, the density of any fiber-reinforced concrete often does not change considerably.

This tendency according to past investigations is the reverse of what we discovered. Fiber has increased based on weight rather than volume in this case. The GPC is influenced by the weight of nylon66 fibers itself. More fiber is added, which boosts the NFRGC’s performance. We employed the dry test, with a total density of GPC of 2400 kg/m^3^, in this experiment. The sample illustrates that the increase in the fiber addition reduces the shrinkage that can cause the weight loss.

## 4. Conclusions

The purpose of this study was to determine the effect of varying the percentage of nylon66 fiber in fly ash geopolymer concrete on strength performance. Furthermore, chemical, physical, and mechanical testing were performed for evaluating fiber properties, raw material characteristics, and geopolymers. Based on the analysis and experimental data results, the following conclusion can be drawn:

1. The majority of the geopolymers’ basic structure is made up of Si-O-Al, indicating the significance of the Si and Al components in creating strong strength development. The presence of Mg in the geopolymer, on the other hand, hindered the geopolymers’ ability to gain strength. This has disrupted the Ca-Si-O-backbone Al structure, reducing the geopolymers’ ability to produce strength.
2. Geopolymers reinforced by nylon66 fiber exhibit negative data. The interfacial connection between the matrix and fiber is weak due to the plastic’s smooth or hydrophobic surfaces, and the fiber cannot stop the spread of cracks in geopolymers. However, some geopolymer matrix spaces containing plastic fibers fill the spaces between fly ash particles with beads to provide interlocking strength and contribute to good strength. More than 0.50% fiber insertion disrupts the CASH bonding in the geopolymer matrix and reduces its compressive strength.
3. Solid to liquid ratio 2.0, alkali activator 2.5, and 12 M NaOH alongside the aggregate ratio were found to be an optimized combination for the mixture process and molding.
4. NFRGC results show that 0.5% fiber addition yields the best results for 28 days (67.7 MPa) and 90 days (70.13 MPa). Due to the development of the geopolymer itself, 90 days NFRGC shows better data than 28 days. In addition, the properties of geopolymers are affected by their curing time. Figure 7 also shows evidence that increasing the volume of fiber increases energy absorption, which aids in the reduction of crack propagation and fracture before they fracture. The ICPB diamond-shaped nylon66 fibers helps to control crack propagation and reduce crack or fracture on the NFRGC by holding the aggregate and matrix together.
5. For the NFRGC, 0.5% was concluded to be the optimum addition due to the flexural strengths obtained for 28 days and 90 days (4.43 MPa and 4.99 MPa, respectively). Addition of plastic fiber at excess of 0.5% reduces the flexural strength. Short fiber showed a small contribution to the compressive and the young modulus of fibers but improve the energy absorbed, based on Figure 9, the comparison between GPC and NFRGC during the flexural test. Due to the higher volume of fiber friction, GPC fractures at the first crack and NFRGC fractures at the final crack. The dominant mode of fracture for nylon66 fibers is no pull out.
6. Based on Figure 9, the contribution of additional fiber improves the crack propagation and slows the fracture process by changing the process from major to minor crack propagation.
7. The water absorption of geopolymer concrete increased as fiber additions increased. The highest water absorption was obtained for geopolymer concrete with the addition of plastic fibers at 2.0% (0.057), and the lowest was obtained at 0.5% with a value of 0.032. This is due to a decrease in workability caused by the addition of nylon66 fibers, which resulted in an increase in pore formation. Water absorption is low in comparison to 0.5% and plain GPC. When comparing water absorption between 0.5% addition and plain GPC, a small range was found, but a larger range was observed when comparing to other volume fiber ratios. This is due to the variety of fiber shapes available, including cylindrical and diamond, as well as the uncontrollable fiber arrangement inside the geopolymers, which resulted in variations in water absorption. Furthermore, since nylon66 fiber is resistant to corrosion, it did not significantly affect the NFRGC.
8. The slump of geopolymer concrete with nylon66 fiber addition decreased as the plastic fiber additions increased. Increasing the fiber content increases the difficulty of relative movement between the coarse aggregates and motion of the mixture, resulting in less workability and flow. The main point is that ICPB diamond-shaped fiber contributes to low workability and a higher viscosity of the NRGPC, and holds the aggregates, giving high resistance in moving the mixture.
9. The change in density value with the addition of fiber in geopolymer concrete does not reveal any discernible trend in which the density rises, because the fiber also has its own density, which may lead to an increase of density of NFRGC.
10. There needs to be more study in these fibers with different materials and dimensions.

## Figures and Tables

**Table 1:** Mix design of nylon66 fiber reinforced geopolymer concrete for compressive.

Plastic Fiber Addition (Kg/m^3^)	Fly Ash (kg/m^3^)	Coarse Aggregate (kg/m^3^)	Fine Aggregate (kg/m^3^)	Sodium Silicate (kg/m^3^)	Sodium Hydroxide (kg/m^3^)
0	640.00	864.00	576.00	229.00	91.00
0.012	630.44	851.10	567.40	225.58	89.42
0.024	620.99	838.34	558.89	222.20	88.30
0.036	611.63	825.70	550.47	218.85	86.97
0.048	602.36	813.19	542.13	215.53	85.65

**Table 2:** Mix design of nylon66 fiber reinforced geopolymer concrete for flexural.

Plastic Fiber Addition (kg/m^3^)	Fly Ash (kg/m^3^)	Coarse Aggregate (kg/m^3^)	Fine Aggregate (kg/m^3^)	Sodium Silicate (kg/m^3^)	Sodium Hydroxide (kg/m^3^)
0	3200.00	4320.00	2880.00	1145.00	455.00
0.06	3152.20	4255.50	2837.00	1127.90	447.10
0.12	3104.95	4191.70	2794.45	1101.00	441.50
0.18	3058.18	4128.50	2752.35	1094.25	438.85
0.24	3011.80	4065.96	2710.65	1077.65	428.25

**Table 3:** Chemical composition of fly ash.

Composition	Weight%
${\mathrm{SiO}}_{2}$	30.80
CaO	22.30
${\mathrm{Fe}}_{2}O_{3}$	22.99
${\mathrm{Al}}_{2}O_{3}$	13.10
MgO	4.00
$K_{2}O$	1.60
${\mathrm{TiO}}_{2}$	0.89
${\mathrm{SO}}_{3}$	2.67
MnO	0.21
Others	1.44

## Data Availability

Not applicable.
